# A rewiring model of intratumoral interaction networks

**DOI:** 10.1016/j.csbj.2019.11.009

**Published:** 2019-11-30

**Authors:** Mengmeng Sang, Shawn Rice, Libo Jiang, Xin Liu, Claudia Gragnoli, Chandra P. Belani, Rongling Wu

**Affiliations:** a Beijing Advanced Innovation Center for Tree Breeding by Molecular Design, Center for Computational Biology, College of Biological Sciences and Technology, Beijing Forestry University, Beijing 100083, China; b Penn State Hershey Cancer Institute, Penn College of Medicine, Hershey, PA 17033 USA; c Department of Medicine, Penn College of Medicine, Hershey, PA 17033 USA; d Methodist Hospital Division of Thomas Jefferson University Hospital, Philadelphia PA 19107 USA; e Department of Public Health Sciences, Pennsylvania State University College of Medicine, Hershey, PA 17033 USA

**Keywords:** Intratumor heterogeneity, Game theory, Community ecology, Cellular interactions

## Abstract

Intratumoral heterogeneity (ITH) has been regarded as a key cause of the failure and resistance of cancer therapy, but how it behaves and functions remains unclear. Advances in single-cell analysis have facilitated the collection of a massive amount of data about genetic and molecular states of individual cancer cells, providing a fuel to dissect the mechanistic organization of ITH at the molecular, metabolic and positional level. Taking advantage of these data, we propose a computational model to rewire up a topological network of cell–cell interdependences and interactions that operate within a tumor mass. The model is grounded on the premise of game theory that each interactive cell (player) strives to maximize its fitness by pursuing a “rational self-interest” strategy, war or peace, in a way that senses and alters other cells to respond properly. By integrating this idea with genome-wide association studies for intratumoral cells, the model is equipped with a capacity to visualize, annotate and quantify how somatic mutations mediate ITH and the network of intratumoral interactions. Taken together, the model provides a topological flow by which cancer cells within a tumor cooperate or compete with each other to downstream pathogenesis. This topological flow can be potentially used as a blueprint for genetically intervening the pattern and strength of cell–cell interactions towards cancer control.

## 1 Introduction

Cancer is composed of genotypically and phenotypically heterogeneous cells that are distributed spatially within the tumor, presenting a phenomenon called intratumoral heterogeneity (ITH) [1], [2], [3]. Because of this complexity, cancer pathogenesis is not only determined by the amount of the constituent cells, but also depends on how these cells interact with each other and with their microenvironment in the body [4]. Currently used genome-wide association studies (GWAS) that sample a set of patients from a natural population have been instrumental for characterizing key somatic mutations that cause tumor growth [5], [6], [7], [8], but these genes identified are merely associated with the overall symptom of cancer, not with the pattern and strength of ITH [9]. Also, genes derived from patients’ normal tissues by GWAS may be related with cancer progression through a myriad of indirect pathways, rather than directly participate in this event as derivers [6].

To identify direct drivers responsible for ITH, it is crucial to reveal what general rules govern its structure and organization. Cancer is actually an ecosystem composed of distinct cell subpopulations that are the equivalence of asexually reproductive, unicellular quasi-organisms [10] and, therefore, its evolution can be understood by Darwin’s natural selection principle [1], [11], [12]. By communicating with each other via hormones, oxygen and nutrients, one cell subpopulation may affect the behavior of other subpopulations both locally and at a distance. The pattern of such a communication can be competitive (like a “war” by which each subpopulation grows at the cost of others) or cooperative (like a “peace” by which different subpopulations can benefit from one another), depending on many factors including the geographic distribution of cells and somatic mutations [12], [13]. Rapid advance in single-cell analysis provides a possibility to genome-wide genotype, sequence and phenotype intratumoral cells spatially located at different positions within a tumor [14], but the identification of ITH-related war and peace and their underlying mechanisms from these data remains a major challenge.

Here, we develop a novel model to investigate the internal workings of ITH. This model is grounded on the principle of game theory integrated with ecosystem theory through computational modeling, thus equipped with a capacity to quantify and interpret how cells interact with each other ecologically within a tumor and how genetic mutations rewire up one cell to others geographically distributed at different places of the tumor. Ecosystem theory aims to study the emergent property of a system mediated by its interconnected components [15], whereas game theory strives to identify a rational strategy chosen by each individual for its maximum payoff [16], [17]. This strategy is called “rational” because its choice both relies on and can determine the strategies of other members in the game. Our unified model can discern the competitive and cooperative pattern of cell–cell interactions that occur pervasively within a tumor. By analyzing the association between (epi)genetic variants and cancer phenotypes or endophenotypes, the new model enables the detection and mapping of cancer-susceptibility genes that are expressed specifically in response to the microenvironment of a given patient.

## 2 A model for mutation identification

### 2.1 Cancer as an ecosystem

The concept of ecosystem was introduced into cancer research four decades ago [1], [4], [18]. This pioneering thinking has well been confirmed by current next-generation sequencing. The cellular composition of cancer is highly heterogeneous with divergent lineages of transformed cancer cells that evolve into metastasis through different pathways and at different rates [18], [19]. Such ITH is one of the primary reasons why current several major therapeutic approaches, such as surgery, chemotherapy and radiotherapy, cannot treat and eradicate cancer completely [20]. A therapy may kill one dominant population of cancer cells, but it is powerless to some other minor populations that, after the treatment, grow and become dominant [21]. Thus, even if successive interventions that often accompany substantial toxicity to patients are used, cancer would still recur at particular times.

On the other hand, cancer cells do not divide, proliferate and metastasize in isolation, instead they have evolved sophisticated strategies for their intimate cooperation and coordination to achieve maximum fitness in a given host environment [2], [3]. Using the classic MMTV-Wnt1 mouse mammary tumor model, Cleary et al. [22] observed that breast cancer can propagate only when its two genetically distinct subclones, luminal *Wnt1*^high^*HRas*^wild-type^ and basal *Wnt1*^low^*HRas*^mutant^, are transplanted together into mammary fat pads of wild-type host animals. This study convincingly shows that cell–cell cooperation is a key driver of breast cancer growth. Thus, by uncoupling such cooperation of different types of cells by designing and delivering pharmacological intervention, cancer can be well controlled [3].

### 2.2 Oligogenic model of ITH

Traditional GWAS through a direct link of patient’s DNA to cancer risk does not capture the mechanistic underpinnings of how internal workings occur within a tumor. It has been evidenced that cancer is initiated due to point mutations or other genetic alterations on particular regions of the genome [23], [24]. Some mutations in a particular cell confer a selective advantage for this cell to outgrow the cells that surround it, forming a patch of clones. Such mutations, called “driver” mutations, are believed to play a key role in the evolution of tumor from benign to malignant lesions. Relative to driver mutations, there are also “passenger” mutations that have no effect on the neoplastic process. It is of fundamental importance in clinical therapies to distinguish the drivers from the passengers although this is usually not an easy task. Other alterations that cause cancer include copy-number alterations, translocations and epigenetic changes.

**Fig. 1:**
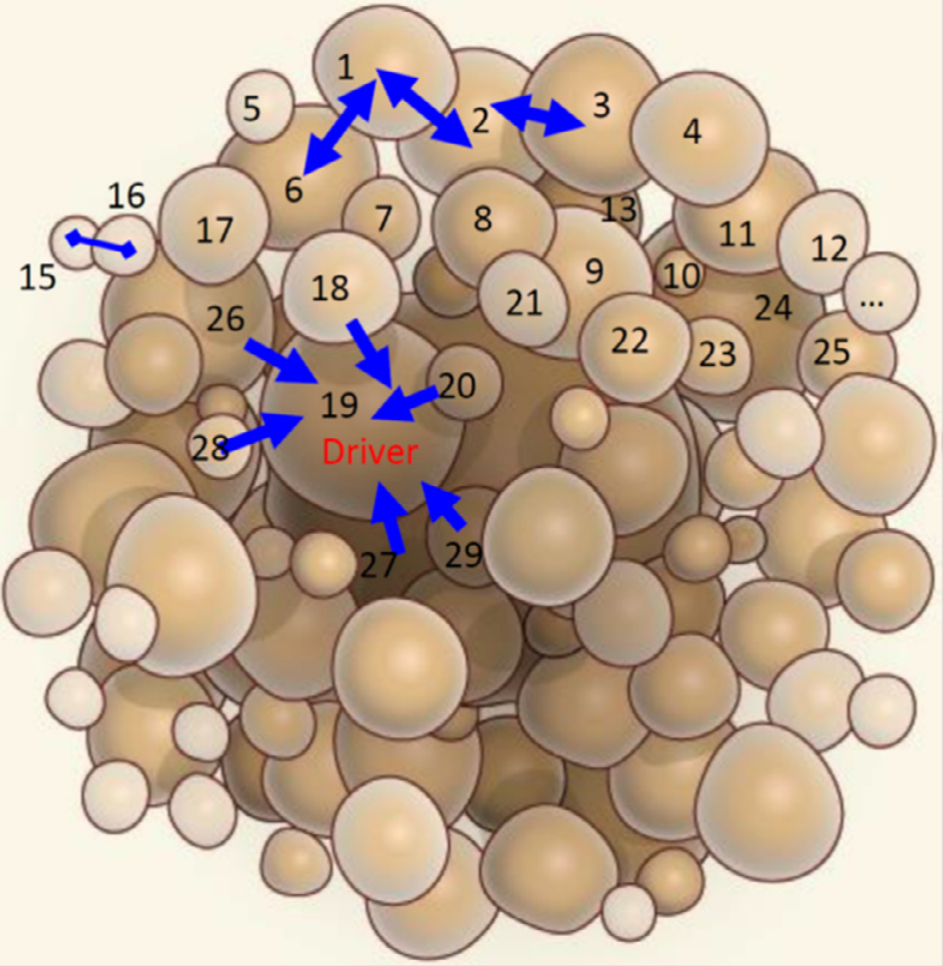
Topological landscape of a tumor. Some cells are labeled for explanation. Two-way arrows, one-way arrows and a line ended with two diamonds denote cooperation, parasitism and competition, respectively. Adapted from Komarova [36].

More recently, increasing studies show that many drivers may provide a relatively modest selective advantage, in contrast to a few drivers of large effects on cancer development [25]. This so-called oligogenic model of drivers is analogous to the genetic control mechanism of many quantitative traits [26]. From this standpoint, cancer cells can be viewed as individual quasi-organisms with continuously varying phenotypes modified by many minor genes and environment. This line of idea is illustrated by a diagrammatic tumor (Fig. 1), in which some cells are large, some are small, whereas most are intermediate from small to large. Mutations that cause the quantitative variation of cell phenotypes or endophenotypes can be identified by genotyping and phenotyping individual cells. However, classic quantitative genetic theory is insufficient to explain such phenotypic variation among cancer cells, because there exists noted cell–cell interdependence. To take into account the phenomenon that no organism may live in isolation, Zhu et al. [27] developed a general framework that integrates game theory into quantitative genetic theory in a way that enables the estimation of how an individual’s phenotype is jointly controlled by its own genes, the genes from its conspecific, and epistatic effects between genes derived from two interactive individuals.

### 2.3 GWAS for intratumoral cells

Today’s genome-wide analysis through sequencing of the exome or whole genome allows all of genes altered in cancer to be characterized at base-pair resolution [14]. These techniques can not only genotype and sequence individual cancer-cell subpopulations, but also dissect the spatial structure and organization of a tumor [28]. In Fig. 1′s hypothesized tumor spherically populated by a number of abundance-varying cell populations, variation in cell abundance may be attributed to different types of mutation. For example, cell 19 is strikingly larger than cell 20, suggesting that the former contains specific mutations that leads it to grow faster than the latter. With no doubt, the genome-wide identification of such mutations helps our understanding of the genetic mechanisms underlying the bewildering diversity of cancer cells.

Box 1 provides a statistical procedure for identifying the mutations that govern the growth and variation of cancer cells. Based on the geographic distribution of cancer cells (Fig. 1), we randomly sample a set of representative cells to measure their mutations throughout the entire genome and phenotypes (such as cell abundance) or endophenotypes including transcriptomic, proteomic, and metabolic profiles by single cell analysis. With the advance of single-cell sequencing techniques, it has become highly possible to measure time series data for the phenotypes. Genome-wide association studies (GWAS) of mutations and cell phenotypes by which to characterize drivers for cell growth can be performed by several statistical methods. They are single-mutation analysis based on simple regression, epistatic analysis aimed to detect significant genetic interactions and high-dimensional variable selection for analyzing all mutations at a time. The first two models are based on the marginal effects of individual loci or locus pairs, thus neglecting the confounding effect of correlated mutations. The third model can make a global characterization of all possible significant mutations whose statistical properties have been validated through empirical data analysis and computer simulation [29], [30].

Box 1: Statistical design and algorithm of cancer genome-wide association studies for intratumoral cells To make it useful in practice, we implemented a statistical procedure for materializing the new theory that is the unification of ecosystem theory and game theory. Using the structural distribution of cancer cells in Fig. 1 as an example, suppose that we have measured mutation loci of each labelled cell and its phenotypes or endophenotypes including transcriptomic, proteomic, and metabolic profiles by single cell analysis. It is possible that time series data for these phenotypes can be made available with the advance of single-cell sequencing techniques. To make a comparison, these types of information are measured for normal cells around the tumor. Below is the hypothesized format of data measured for labeled cancer cells and normal cells:

**Figure:**
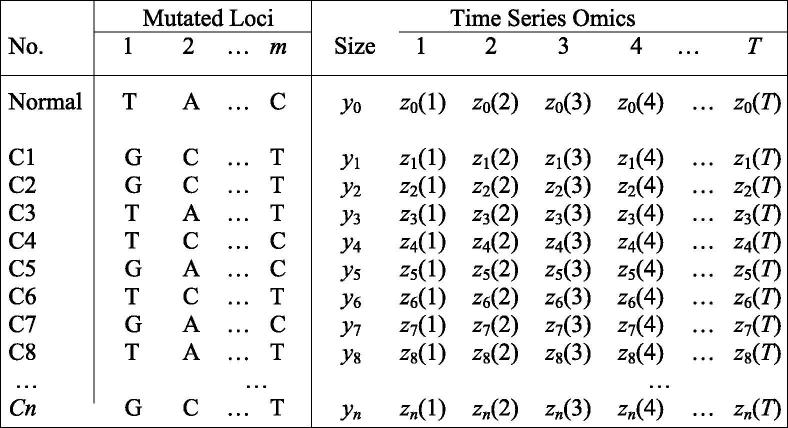

To reflect the tenet of the new theory, i.e., different cells interacts to reciprocally affect their phenotypes, we arrange the data by physical connections of different cells. For example, cell population 1 is linked with cell populations 2 and 5, which suggests that the former should be paired with the latters. Based on this principle, we obtain the new data format as follows:

**Figure:**
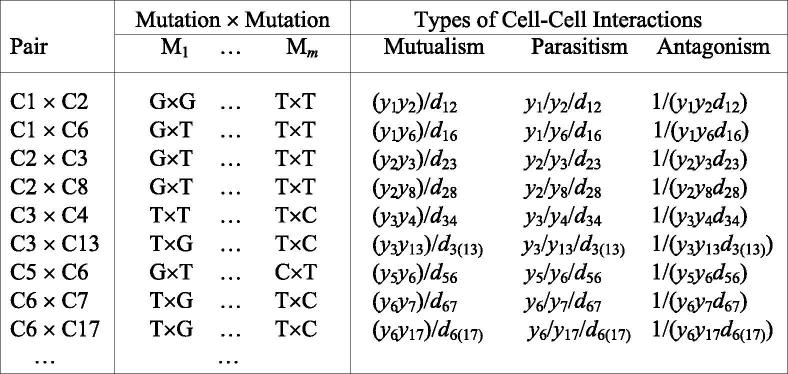

where *d..* is the physical distance between different cancer cells. The phenotypes of adjacent cells (i.e., cancer size) are also paired in terms of their competitive or cooperative relationships. To characterize how genetic mutations determine cancer phenotypes through cell–cell heterogeneity and interactions, we formulate the likelihood of the above re-arranged data, which, for any mutation locus (say M_1_), is expressed as.

**Figure:**
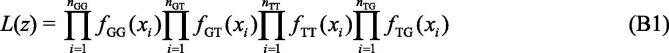

where cell–cell pairs form four different mutation combinations G × G, G × T, T × T and T × G at this locus; *n*_GG_, *n*_GT_, *n*_TT_, and *n*_TG_ are the observations of four mutation combinations, respectively; and *f*.(*z_i_*) is the probability density of a derivative variable *x* (denoting a product, ratio, or the inverse of product; see the second data format), with expected mean $\mu_{\cdot }^{x}$ for a particular combination and variance σ^2.^

By estimating and testing the differences of the expected mean between these combinations, we can determine whether this mutation affects the derivative trait. Zhu et al. [27] developed a method for dissolving the genetic effects of the derivative variable into direct, indirect and epistatic effects on the original phenotypic trait of two pairing partners. Here, the direct effect denotes the genetic effect of a mutation of a target cell on its own phenotypic trait, the indirect effect implies the genetic effect of the mutation of this target cell on the phenotypic trait of its partner, and the epistatic effect is the across-cell genetic interaction effect of the mutations derived from the two paired cells on the phenotypic traits of the two cells. Zhu et al. [27] also provided a procedure of testing each of these effects based on a likelihood-ratio approach.

We extend Zhu et al.’s procedure to estimate the genetic variances of the phenotypic trait of the target cell by the direct effect, indirect effect and across-cell epistatic effect, respectively. From these estimates, we can further estimate the proportions of the total genetic variance by each of these effects. This proportion describes the relative contributions of direct, indirect and across-cell epistatic effects to the total phenotypic variance.

### 2.4 Worked example

**Fig. 2:**
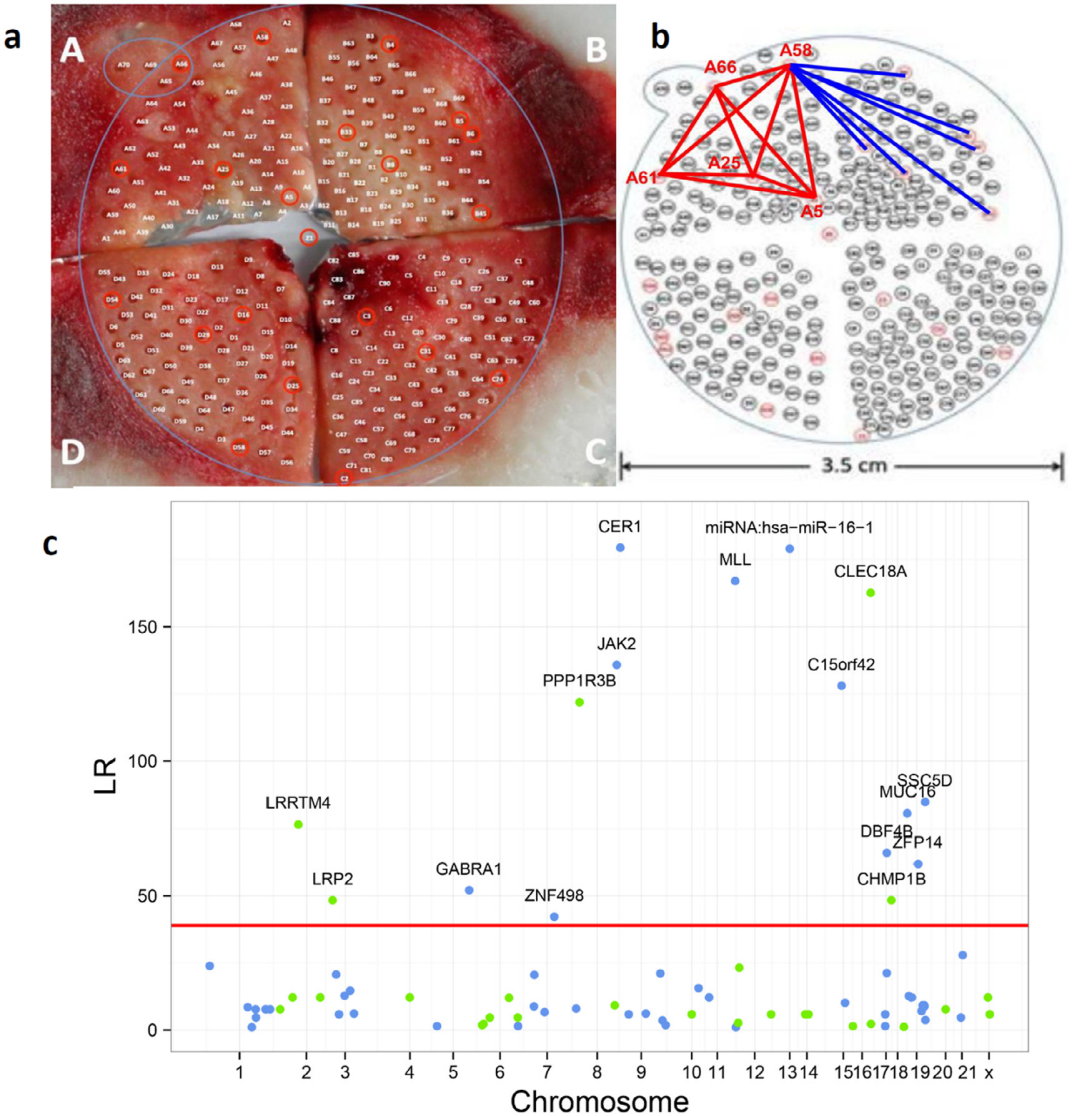
Sample collection and data analysis for an HHC tumor [31]. (**a**) Honeycomb-like microdissection. (**b**) Geographic locations of 286 regions, 23 of which (in red) were monitored in detail. We connected some regions to show their interactions. (**c**) Manhattan significance test plot of 87 segregating loci that affect cell–cell variation in ploidy level. LR is the log-likelihood ratio test derived from the likelihood (Box 1). Horizontal line is the genome-wide threshold at the 1% level determined from 1,000 permutation tests. (For interpretation of the references to colour in this figure legend, the reader is referred to the web version of this article.)

To demonstrate the utility of our model, we reanalyze a published data of cancer collected by high-throughput sequencing. As a conceptual idea, our model can characterize intratumoral interactions that occur at any level of entities, from cell, cell population to geographical region. In a single hepatocellular carcinoma (HCC) tumor, Ling et al. [31] idebtified 286 representative spatial regions, of which 23 were genotyped at 269 mutation loci over 19 human chromosomes and phenotyped (Fig. 2a). Each region may contain a specific cell population and thus, our analysis will focus on intratumoral interactions at the cell population level. We use ploidy levels of each region as a phenotypic trait for intratumoral GWAS analysis. The level of ploidy is thought to be an indicator of cancer pathogenesis [32] so that we can use it as a fitness-related phenotype of cancer. The tumor is divided into four quadrants A–D, from each of which 23 regions were randomly and evenly sampled (Fig. 2b). Of 269 genotyped loci, 87 are segregating in 23 regions, which are associated with ploidy level through the analysis of likelihood (Box 1). It can be seen that genetic mutations occur stochastically over 23 human chromosomes. Of 87 polymorphic loci among the sampled cell populations, 16 are significantly associated with the level of cell ploidy (Fig. 2c). Several significant mutations, such as CER1, miRNA:has-miR-16–1, and MLL, each explain 10–15% of the phenotypic variation, remarkably larger than many others. These loci are the drivers that cause the heterogeneity of intratumoral cells.

## 3 Computing cooperation and competition within a tumor

As can be seen from a tumor (Fig. 1), cell populations 1, 2, 3 and 6 are equally large, possibly implying their involvement of growth-promoting mutations. Another possibility is that these cells mutually cooperate to favor their growth and proliferation given their geographic adjacency. On contrast, two adjoining populations 15 and 16 are small-sized, possibly due to their specific mutations and mutual conflict. Cell population 19 is much larger than its neighboring populations, 20, 29, 18, etc., suggesting that the latter is parasitized by the former. However, precisely characterizing these competitive (war) or cooperative (peace) types of cell–cell interactions, as a stepping stone to cancer diagnosis and therapy, is very challenging and requires a conceptual theory and modeling framework.

### 3.1 Game theory

In a tumor, different populations “live” together following Darwin’s rule. Consider two cell populations A and B, each of which tends to maximize its fitness under Darwinian selection using a cooperative (+), spite (–) or neutral (0) strategy [33]. If both populations are cooperative, they benefit from each other. If each population spites its partner, then both grow at a competitive cost of the counterpart. If one is cooperative but the second gives spite, then only the latter benefits in an altruistic or parasitic way. It is not difficult to see that the fitness of any cell population in its interconnected environment is the net consequence of competition, cooperation and parasitism. According to evolutionary game theory, these interaction patterns can be formulated by a *strategy matrix* expressed as

**Table:** 

		Population B			
		+	0	–	(1)
Population A	+	Mutualism	Commensalism	Predation	
	0	Commensalism	Coexistence	Amensalism	
	−	Predation	Amensalism	Antagonism	

Different strategies used by each cell population will form several types of ecological interactions [2], [4]: **mutualism** (+/+) by which two populations cooperate and produce factors or bring in resources that will benefit both interacting parties, **peaceful coexistence** (0/0) by which the two populations can co-exist but have no dramatic benefit to each other, **antagonism** (–/–) as the strongest negative interactions arising due to limitation in resources such as nutrients and oxygen, which can manifest phenotypically via the secretion of molecules by one population that either kills or suppresses competitor cells and vice versa, **commensalism** (+/0 or 0/+) by which one population can benefit another without being affected itself, **predation**/**parasitism** (+/–) that benefit one population by consuming biomass at the expense of the other, although predation is more extreme than parasitism, and **amensalism** (0/– or –/0) that is similar to competition but occurring more undirectionally, involving the inhibition of one population by another with the latter being not affected.

### 3.2 A computing framework

Coordinated cooperation and competition among cancer cells of differing genetic makeup have important consequences for the overall behavior of cancer. By combining network analysis and game theory, a cell-rewiring model was developed to study, discern and annotate war and peace that occur within the communities of cancer cells. The model was further reformed to unravel the genetic architecture that govern the direction and strength of cell–cell interactions. Let *y*_1_ and *y*_2_ denote the values of a fitness-related trait for two co-occurring cell populations A and B, respectively. The product of *y*_1_ and *y*_2_ is used to measure the extent of their **cooperation**; their ratios reflect the extent of the benefit of one population relative to the other (**altruism**); and the inverse of their product measures the extent of **competition**. In Box 1, following Zhu et al.’s [27] game-analytical model, we describe a likelihood model for characterizing these interactions and the genetic machineries underlying each interaction.

### 3.3 Application

**Fig. 3:**
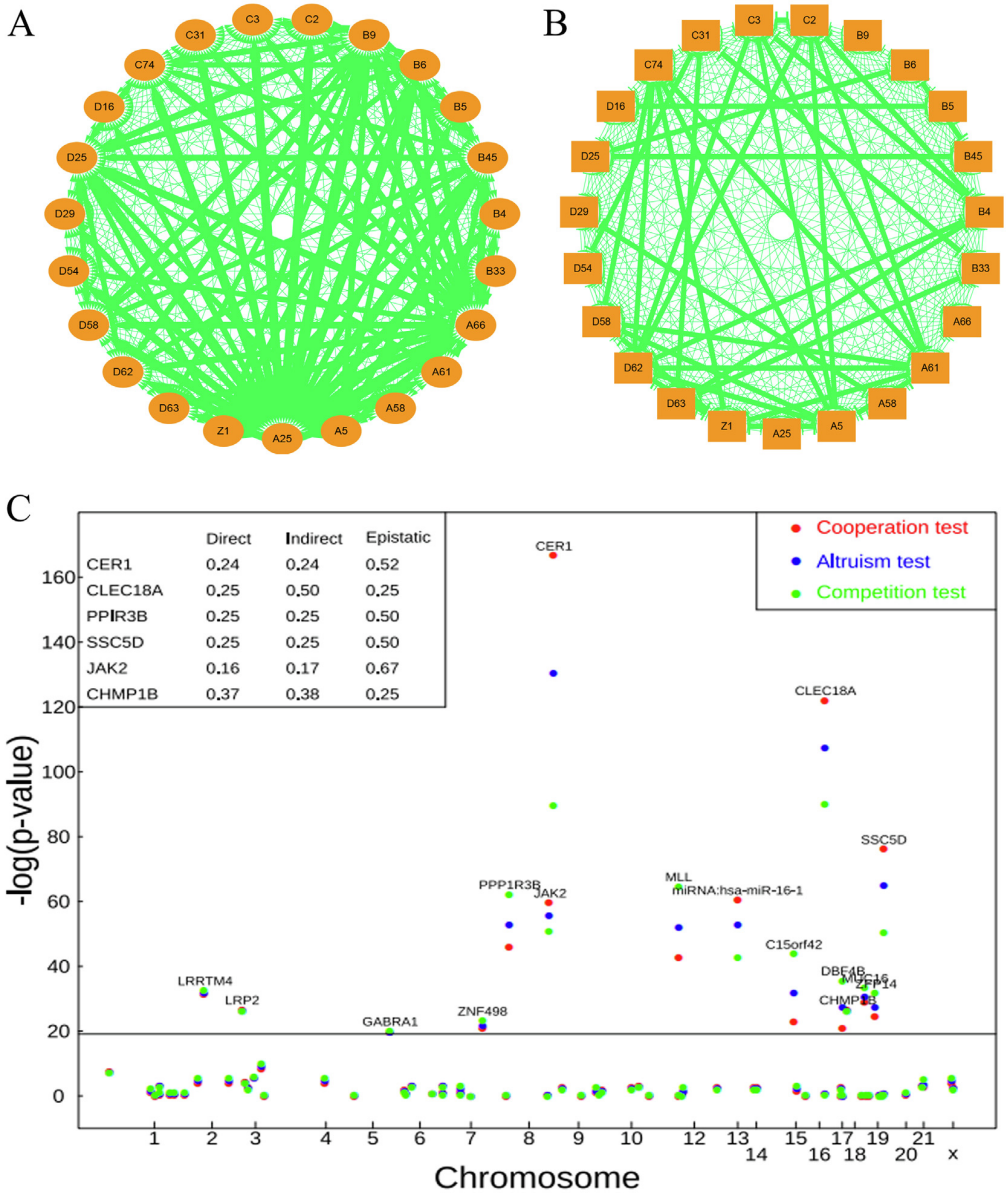
The networks of cooperative interactions (**A**) and competitive interactions (**B**) among 23 sampled cell regions constructed by game theory. Dually arrowed lines indicate the mutual activation of two cell regions, whereas T-shaped lines represent the inhibition of one cell regions by another. Manhattan significance test plot of genetic mutations for cell–cell cooperation (red), cell–cell altruism (blue) and cell–cell competition (green) through ploidy level within the HHC tumor (**C**). LR is the log-likelihood ratio test derived from the likelihood (Box 1). Horizontal line is the genome-wide threshold at the 1% level determined from 1,000 permutation tests. Numbers in the box at the upper left are the portions of the genetic variance explained by the direct effect, indirect effect and across-cell epistatic effect of six representative mutation loci. (For interpretation of the references to colour in this figure legend, the reader is referred to the web version of this article.)

By analyzing sequence data from individual regions within the HCC tumor (Fig. 2A), Ling et al. [31] found the existence of intratumoral genetic diversity, although it does not obey Darwinian evolutionary theory. Our model can identify whether intratumoral genetic diversity results from interactions between different cell populations. Twenty-three randomly sampled regions were paired to quantify their cooperative, altruistic and competitive interactions in terms of ploidy level, weighted by their pairwise distances. Overall, the cooperative network (Fig. 3A) contains much more connections than does the competitive network (Fig. 3B), suggesting that cell–cell cooperation is more pervasive within this tumor studied, compared to cell–cell competition. It can be seen that region A25 strongly cooperates with the largest number of other regions, followed by A66, B9 and D25. These regions connecting with so many other regions, called hub regions, play a central role in maintaining the stability of cell–cell interaction network (Fig. 3A). Uncoupling the connections of these hub regions with other regions likely causes the instability of the cell community and, therefore, affects the holistic behavior of cancer. Although connections are much sparser in the network of competition (Fig. 3B), several regions can still be identified to repress the expression of other regions.

The cell-rewiring model was further used to identify 16 significant mutation loci distributed in different chromosomes that regulate intratumor interactions between these regions (Fig. 3C). Each of these loci pleiotropically affects cooperation, altruism, and competition, although some loci exert a larger effect on one type of interaction than others. For example, LRRTM4, LRP2, GABRAl1, and ZNF498 each exhibit a similar magnitude of their effect on the three types of interactions, CER1, CLEC18A, SSC4D, miRNA:hsa-miR-16–1 and JAK2 play a larger role in modulating cooperation than competition, and other loci, such as PPP1R3B and MLL, are determinants of cell–cell conflict in the first place and determinants of cooperation in the second place.

In addition to identifying how a mutation regulates the fitness of cancer cells, the model can provide unique insight into the genetic machineries of cell–cell interactions. For example, the effect of a mutation on intratumor interactions can be further partitioned into three components, i.e., the direct effect of the mutation, carried by a target cell, on the own fitness of this cell, the indirect effect of this mutation on the fitness of the other cells that are geographically close to the target cell, and the across-cell epistatic effect of the mutation derived from a different cell (Box 1). We estimated the portions of the total genetic variance explained by the indirect effect, indirect effect and across-cell epistatic effect for each significant locus as a cooperation gene, altruism gene or competition gene, respectively. It is interesting to find that the indirect effect contributes to genetic variance as much as the direct effect, both generally accounting for 20–30%. Surprisingly, the across-cell epistatic effect explains a large portion of genetic variance, which, in most cases, is even larger than those explained by the direct effect. Beyond all existing approaches, our model can dig out the hidden genetic architecture of cancer phenotype by identifying indirect and across-cell epistatic effects or variances.

## 4 Discussion

As a complex disease, cancer is jointly controlled by genetic and environmental factors and their interactions through developmental pathways. A vast body of cancer research has used genome-wide association studies (GWAS) to identify genetic loci associated with cancer risk by comparing differences of DNA sampled from a natural population [6], [7], [8], [9]. However, despite our mounting knowledge gained from GWAS, an overwhelming success in understanding the genetic basis of this disease has still been largely far from our expectation. We have increasingly recognized that this failure results largely from a high ITH, characterized by a tumor, but the systematic identity of individual cell states has not been made possible until the advent of single-cell sequencing techniques. How to transform increasingly growing amounts of single-cell data into a procedure of predicting the mechanistic and regulatory programs of cancer development has now become one of the most important tasks.

In this article, we present a novel model that unifies ecosystem theory and game theory to model, annotate and contextualize internal workings within a tumor driven by cancer cells. Although genetic ITH has made it extremely difficult to remove cancer, the interdependence of cancer cells may open up a new avenue to control this disease. For example, if cancer growth relies on the cooperation of its cells, then a particular pharmacological intervention can be designed and delivered to uncouple this cooperation. If different cells compete for the same type of resources such as oxygen and nutrient, specific drugs can be developed to induce and amplify their conflict, leading to the simultaneous eradication of these cells. Apart from internal interactions within cancer, cancer cells also interact with the tissue environment of the host; for example, immune cells may predate and, ultimately, destroy cancer cells. This process has led to the birth of immunotherapies that control cancer by increasing the patient’s immune capacity [34].

It is possible that the exploitation of cell–cell interrelationships can give rise to a new cancer therapy that is different from, but likely to be more effective and efficient in terms of the prevention of recurrence and side effects, than currently used surgery, chemotherapy and radiotherapy approaches [35]. The first most important step to make this assumption a reality is to profoundly understand how intratumor cells interact internally with each other and how they interact externally with the immune cells of the host. Our conceptual model provides a start point to further chart a detailed atlas of ecological interactions among cancer cells and the host’s immune cells, and could also stimulate the collaboration of oncologists, pharmacologists and statisticians to determine and select an optimal treatment for cancer.

## Author contributions statement

RW conceived of the idea and wrote the paper. MS and LJ performed statistical modeling and testing. SR, XL, and CPB provided insight into intratumoral interactions. CG read and edited the manuscript.

## Declaration of Competing Interest

The authors declare that they have no known competing financial interests or personal relationships that could have appeared to influence the work reported in this paper.
